# From Isolation to Integration: Harnessing Restorative Clinical Supervision Within the A‐EQUIP Model to Support Internationally Educated Radiographers in the UK

**DOI:** 10.1002/jmrs.70128

**Published:** 2026-09-25

**Authors:** Dorothy Cox, Andrea Stebbings

**Affiliations:** ^1^ School of Education, Health and Science University of Gloucestershire Gloucester Gloucestershire UK; ^2^ Department of Radiology Gloucestershire Hospitals NHS Foundation Trust Gloucester Gloucestershire UK; ^3^ School of Nursing and Midwifery University of Plymouth Plymouth Devon UK

## Abstract

Diagnostic radiographer vacancy rates in the UK continue to rise, placing growing reliance on international recruitment to sustain the workforce. Yet, whilst internationally educated doctors and nurses benefit from established transition frameworks, no equivalent exists for internationally educated radiographers. Evidence suggests this group faces compounded professional, personal and cultural stressors on entering NHS employment, contributing to poor wellbeing and high attrition. This commentary examines the literature to explore whether the Advocacy and Education for Quality Improvement in Practice (A‐EQUIP) model of restorative clinical supervision offers a viable support framework for this workforce. A‐EQUIP has demonstrated effectiveness in nursing and midwifery, though its application to Allied Health Professionals remains largely unexplored. Drawing on evidence across radiography, nursing, midwifery and allied health literature, this paper considers transitional stressors against the functions of A‐EQUIP, examines barriers to implementation and offers evidence‐informed recommendations for organisations, managers, professional advocates and professional bodies.

## Graphical abstract

Internationally educated radiographers face isolation, unfamiliar hierarchies and discrimination risk when transitioning to UK practice. Embedding restorative clinical supervision within the A‐EQUIP model offers a practical, evidence‐informed framework to reduce isolation and improve retention within radiography departments.

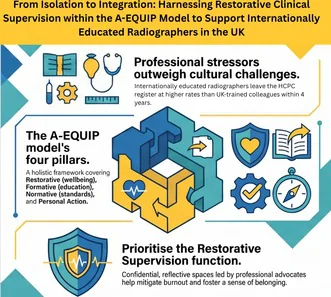

AI statement: The graphical abstract was initially generated using generative AI and subsequently edited and refined by the authors using Canva.

## 1 Introduction

Diagnostic radiographer vacancy rates in England have risen consistently and a global shortfall in the profession has prompted healthcare providers to look increasingly to international recruitment [1, 2, 3, 4, 5]. Demand compounds the pressure; as highlighted in the Diagnostics: Recovery and Renewal report [6], diagnostic imaging tests performed annually in England increased by 24.1% over the last decade [7, 8], with workforce capacity already stretched.

Internationally educated doctors and nurses joining the NHS benefit from structured transition support, including profession‐specific induction programmes and frameworks such as the ‘Welcome to UK Practice’ programme [9, 10, 11]. No comparable provision exists for internationally educated radiographers [9, 10, 11]. A nationally driven AHP preceptorship framework is available [12], but it is not profession‐specific and does not address the particular challenges this group faces. Internationally educated radiographers frequently report feeling isolated and anxious on joining the NHS, with attendant risks to wellbeing and retention [13, 14].

Proctor's 1987 model of Restorative Clinical Supervision (RCS) encompasses three supervisory functions: restorative, formative and normative [15]. This forms the foundation of the Advocacy and Education for Quality Improvement in Practice (A‐EQUIP) model, which adds a fourth function, personal action for quality improvement, and has been shown to reduce stress and burnout across healthcare settings [16, 17, 18]. This commentary focuses primarily on the restorative function, which supports emotional wellbeing and has the strongest evidence base and most direct relevance to the challenges faced by internationally educated radiographers. The formative, normative and personal action for quality improvement functions are acknowledged where pertinent. Although A‐EQUIP is established in nursing and midwifery, its application to Allied Health Professionals (AHPs) has not been systematically explored; this paper examines the available evidence to consider how the model might support internationally educated radiographers in the UK.

## 2 Drivers for Change

The UK lags significantly behind EU comparators in diagnostic capacity, with below‐average MRI and CT scanner provision per million population [19, 20]. Against this backdrop, a 12.1% vacancy rate in England means diagnostic radiographers are already working under sustained pressure, with occupational stress and compassion fatigue worsened by the COVID‐19 pandemic [3, 20, 21, 22, 23]. For internationally educated radiographers, these systemic stressors are compounded by the additional challenges of migration and professional transition [14, 24, 25].

The evidence base in this area is limited [26] and radiographer‐specific research is scarcer still [13, 27]. A recent survey of 226 internationally educated diagnostic radiographers found that 30.2% had experienced workplace bullying, 34.2% reported cultural isolation and over 40% were considering leaving or uncertain about remaining in the UK [27]. A tendency to prioritise recruitment metrics over lived experience has left a significant gap in the evidence available to inform policy and practice [14].

Given this scarcity, this commentary also draws on the wider literature pertaining to internationally educated healthcare professionals [24]. The stressors presented in Table 1 were identified through iterative searching of the literature, using terms including ‘internationally educated’, ‘overseas’, ‘radiographer’, ‘transition’, ‘integration’ and ‘stressors’, until thematic sufficiency was reached. Findings were organised into three domains (cultural, professional and personal) reflecting the dimensions most consistently identified in the literature. Cultural stressors relate to differences in values, norms and social expectations between countries of origin and the UK; professional stressors encompass workplace‐specific challenges; and personal stressors reflect the psychosocial impact of migration and adaptation. Where radiographer‐specific evidence was unavailable, evidence from comparable internationally educated healthcare professionals was used, acknowledging that direct transferability cannot be assumed [24].

**TABLE 1:** Experiences of internationally educated healthcare professionals, synthesised from qualitative evidence [14, 24, 25, 26, 27, 28, 29, 30, 31].

Cultural	Professional	Personal
Culture shock on arriving in the UK. Elderly care may be provided by family in other cultures; this differs from accepted UK healthcare practice. Personal wellbeing rarely emphasised in countries of origin.	Lack of support, education and training. Workplace loneliness and isolation. Unfamiliar techniques, policies and regulations. Different scope of practice. Discrimination and bullying by colleagues or managers. Different professional communication styles. Fewer opportunities compared with UK‐educated nationals. Unfamiliarity with reflective and evidence‐based practice. Technological differences. Occupational stress; expectation of autonomous working. Less formal hierarchical structures than in countries of origin.	Transport and housing challenges. Feeling unwelcome or professionally inadequate. Different social communication styles. Loneliness and social disconnection outside work. Discrimination and bullying in social settings. Emotional and financial stress.

The preponderance of stressors in the ‘Professional’ domain is notable, suggesting that negative cultural presumptions about internationally educated healthcare professionals are largely misplaced and that targeted workplace support could address many of the difficulties this group faces [14]. Where stressors appear across more than one domain, they reflect meaningfully different experiences: workplace loneliness refers to professional isolation within teams, whilst personal loneliness reflects broader social disconnection outside work. Discrimination also manifests differently across domains; professionally through colleague or managerial behaviour in clinical settings and personally through experiences in social contexts outside work. Communication challenges similarly differ. Professionally, they relate to clinical interaction and workplace norms; personally, they reflect the harder task of building social relationships in a new cultural context.

**FIGURE 1:**
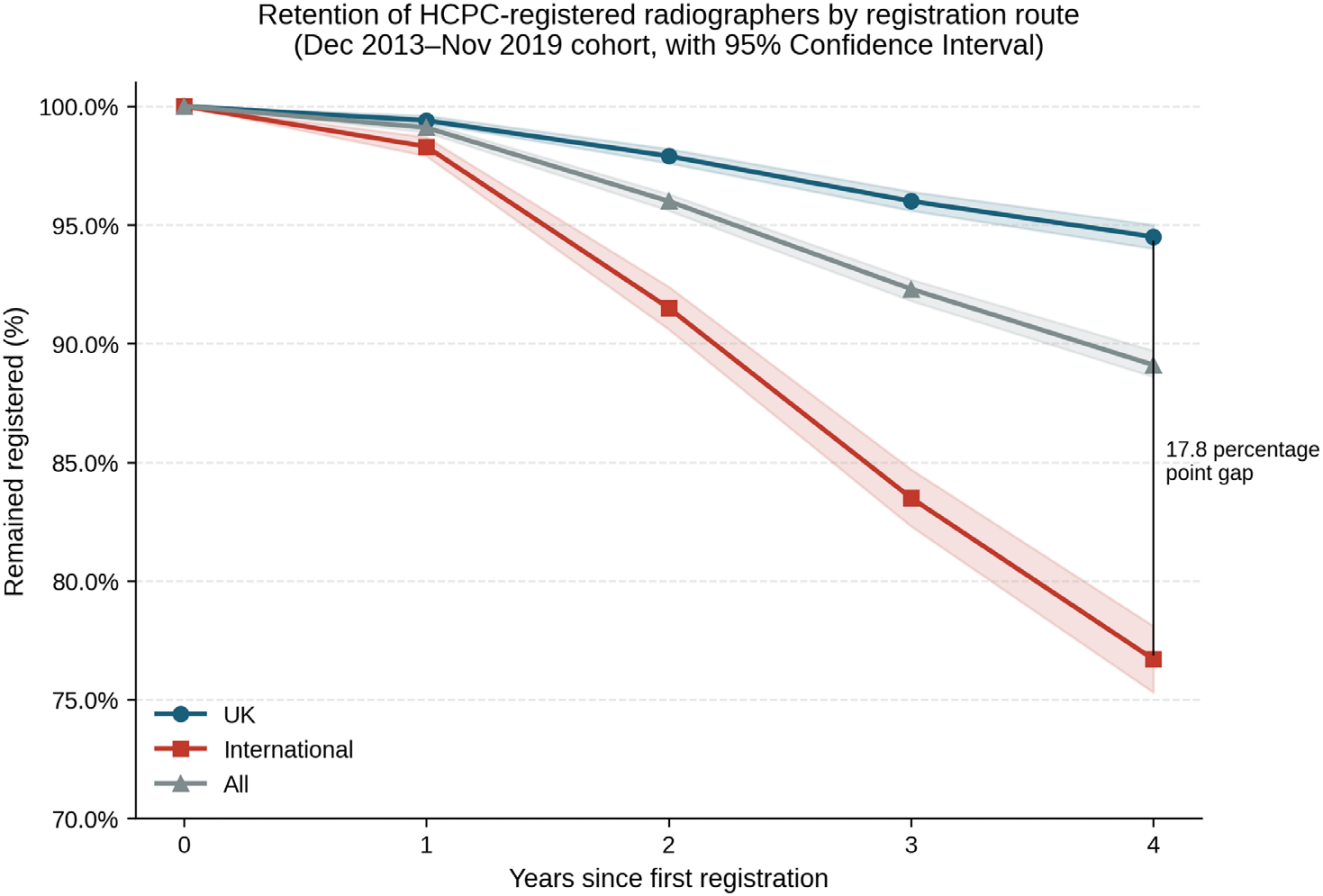
Percentage of HCPC‐registered diagnostic radiographers remaining registered by years since first registration, by registration route (December 2013–November 2019 cohort; shaded bands = 95% confidence intervals). Authors' own analysis and visualisation, based on registration data reported by the Health and Care Professions Council [32].

Professional mentorship improves a sense of belonging, job satisfaction and cultural integration [25, 26, 30], and a pilot programme for internationally educated radiographers in the South West of England demonstrated promising wellbeing outcomes [13]. It is also worth acknowledging that the challenges facing internationally educated radiographers begin before employment. UK registration requires internationally educated radiographers to demonstrate qualification equivalence and meet HCPC proficiency standards [30], adding procedural demands to the transition process. Without adequate support throughout, this accumulation of challenges risks burnout and attrition [14, 24, 31]. HCPC retention data show that by year four, 76.7% of internationally educated radiographers remained registered compared with 94.5% of UK‐trained radiographers, demonstrating a 17.8 percentage‐point gap (Figure 1) [32]. Inadequate educational support regarding UK radiation regulations further risks regulatory disciplinary action, as observed in comparable staff groups [33, 34, 35, 36, 37].

## 3 The A‐EQUIP Model of Supervision

**FIGURE 2:**
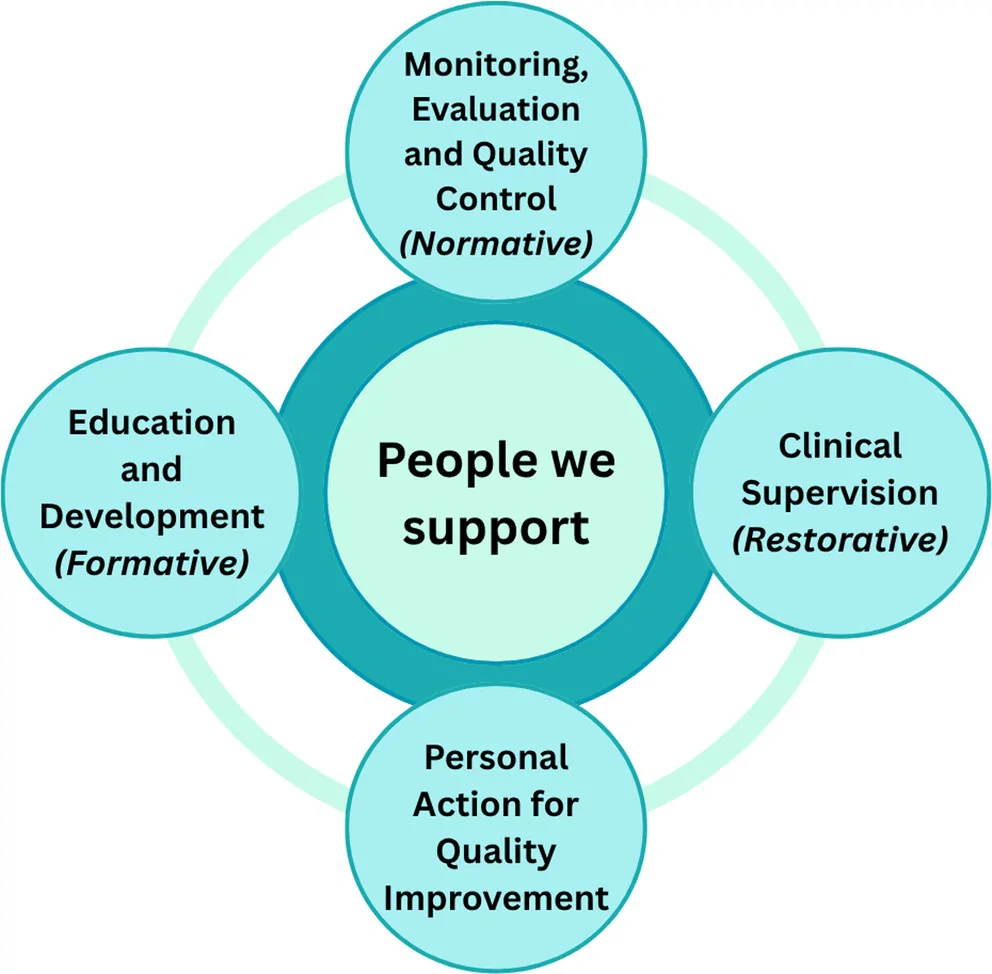
The A‐EQUIP Model [38]. Adapted from NHS England, Professional nurse advocate A‐EQUIP model: A model of clinical supervision for nurses (Version 2, September 2023), https://www.england.nhs.uk/long‐read/pna‐equip‐model‐a‐model‐of‐clinical‐supervision‐for‐nurses/. Contains public sector information licensed under the Open Government Licence v3.0.

The A‐EQUIP model (Figure 2) builds on Proctor's three supervisory functions, namely restorative, formative and normative, by adding personal action for quality improvement, a key professional requirement for radiographers [15, 33, 39, 40]. Professional advocates trained to deliver restorative clinical supervision were first piloted in midwifery in 2017 and extended to nursing in 2021 [38, 41]; AHPs have since been encouraged to engage with the model, with some undertaking the role as part of their substantive position [42]. In practice, professional advocates would be trained members of the AHP workforce who have completed A‐EQUIP education, equipping them with skills in reflective facilitation and non‐directive questioning.

Evidence specifically relating to restorative clinical supervision and diagnostic radiography is scarce; AHP‐focused literature either groups all professions together or excludes radiographers entirely [43, 44], necessitating the use of evidence from other healthcare contexts where appropriately transferable. This limitation is acknowledged throughout and claims about radiographers specifically are distinguished from those drawn from the broader healthcare literature.

### 3.1 Cultural

Fewer stressors were attributed to the cultural domain than to the professional or personal, though this likely reflects the emphasis of the existing literature rather than the true weight of cultural challenges faced by internationally educated radiographers. Culture shock is among the most universally reported. Resilience, understood as the capacity to adapt positively in the face of adversity [45], is often proposed as a response, yet the evidence for education‐based resilience interventions in healthcare is weak [46, 47, 48]. Restorative clinical supervision has shown some promise in improving resilience [49, 50], though a 2018 systematic review raised concerns about absent control groups and the absence of standardised measurement tools [46]. The durability of any improvement also remains unexamined [51].

A more fundamental limitation of resilience‐focused approaches is that they risk placing the burden of adjustment on individuals rather than addressing the organisational conditions that produce culture shock. The professional advocate role within A‐EQUIP is well placed to address this. By creating a reflective space in which internationally educated radiographers can examine their experiences, advocates can bring systemic issues to light and advocate for broader organisational responses such as cultural induction and peer mentoring.

### 3.2 Professional

Internationally educated healthcare professionals may have previously worked within strongly hierarchical systems in which deference to senior colleagues was both expected and enforced [14, 24]. Transposing into the comparatively informal structures of the NHS can be disorienting and may produce a reluctance to speak openly that impedes both professional development and integration. Individual restorative clinical supervision offers a structured counter to this. The professional advocate, operating outside line‐management structures, creates a confidential space in which the supervisee is actively encouraged to reflect, question and develop their own professional judgement without fear of repercussion [40, 52, 53, 54]. Over time, this kind of supported reflection can build the cultural competence and professional confidence that hierarchical conditioning may have suppressed.

Group restorative clinical supervision offers complementary benefits, fostering peer relationships and reducing professional isolation [52, 55, 56, 57, 58], though it requires careful facilitation. Poorly managed sessions can be dominated by more confident participants or feel intrusive to those from cultures where group disclosure is unfamiliar [52, 55, 56, 57, 58]. The methodological limitations of studies in this area, including small sample sizes and brief follow‐up periods, mean that conclusions should be drawn cautiously [55, 56, 57, 58]. Martin et al.'s [52] mixed‐methods systematic review provides the most robust evidence available, concluding that the quality of supervision delivered is the critical determinant of its effectiveness.

The formative, normative and personal action for quality improvement functions support internationally educated radiographers to navigate UK policies, professional standards and regulatory expectations, building confidence and fostering a learning culture. NHS Workforce Race Equality Standard data indicate that staff from minority ethnic backgrounds are significantly more likely to be subject to formal disciplinary processes and to report workplace bullying than their white British counterparts [59]. This distinction matters for this workforce; in a UK‐wide survey of internationally educated diagnostic radiographers, bullying was reported more commonly among respondents from minority ethnic backgrounds than among white respondents [25]. Ethnicity and international educational background are not synonymous, but for those from minority ethnic backgrounds specifically, the challenges of international transition may be compounded by racial discrimination and the implications for radiography departments warrant serious consideration.

### 3.3 Personal

The personal stressors identified in Table 1, namely financial and housing pressures, social isolation, feelings of inadequacy and discrimination, extend beyond what any workplace intervention can directly resolve. What restorative clinical supervision offers is a consistent space in which their emotional weight can be acknowledged and worked through. Stogiannos et al. [45] observe that resilience is not a culturally neutral concept; a standardised programme is unlikely to meet the needs of a diverse internationally educated workforce. Restorative clinical supervision, by contrast, is inherently individualised, as sessions are shaped by the specific concerns and circumstances of each supervisee, enabling the professional advocate to provide support that is responsive rather than formulaic [52, 56]. Through structured reflection, supervisees are supported to articulate their experiences, receive validation and develop coping strategies, promoting self‐awareness and a more secure professional identity [52, 56] and enabling the peer relationships that protect against burnout and long‐term disengagement [22, 24, 52, 56].

## 4 Barriers to Implementation

The case for A‐EQUIP as a support framework for internationally educated radiographers is, on the basis of the available evidence, a plausible and promising one. Acknowledging the barriers to implementation is not a qualification of that argument, but a necessary step towards sustainable adoption. Implementing new ways of working in healthcare is inherently difficult, particularly in services under sustained operational pressure [60, 61], and the barriers here are multi‐level, relating to the organisation, the model itself and the professional advocate role (Table 2).

**TABLE 2:** Barriers to restorative clinical supervision [48, 49, 55, 56, 62, 63].

Organisational	RCS model	Professional advocate
Clinical workload and staffing pressures. Restorative clinical supervision used reactively rather than proactively. Insufficient senior and organisational support. Shift patterns limiting availability. Lack of workforce representation. Staff reluctance to take time away from clinical duties. Absence of suitable space and inadequate advocate‐to‐supervisee ratios.	Perceived lack of fit with practice context. Absence of standardised session frameworks. Over‐reliance on newly qualified staff to promote the model. Poor understanding of what restorative clinical supervision involves.	Inadequate training or skillset. Tendency to adopt a problem‐solving rather than facilitative approach. Supervisee reluctance when the advocate also holds a managerial role. Advocate clinical workload.

Protected time is perhaps the most consistently cited prerequisite for effective restorative clinical supervision [53], yet it is precisely what is in shortest supply in a profession characterised by high throughput, tight operational targets and chronic understaffing [4, 43, 62]. Research on AHP supervision indicates that managerial allocation of dedicated time is one of the strongest predictors of engagement [43]; without it, even the most carefully designed implementation is unlikely to take hold. Blended delivery, including online sessions, can help address access constraints [63].

The composition of the advocate workforce is also important. Internationally educated radiographers are unlikely to engage fully with a supervision process from which people who understand their experience are absent. Representation is a precondition for the psychological safety on which effective restorative clinical supervision depends, not merely a matter of equity. Embedding restorative clinical supervision within existing CPD, appraisal and quality improvement structures, rather than positioning it as an additional demand, reduces both the practical and cultural resistance to adoption [63]. The absence of contractual obligation for radiographers [64], and the age of the SoR's most recent guidance on the subject [65] have left the profession without the structural impetus that has driven implementation in nursing and midwifery. Addressing this at a professional body level would be a meaningful step forward [62].

## 5 Recommendations

Given the absence of radiography‐specific research on A‐EQUIP, the following recommendations are evidence‐informed proposals rather than directives, and primary research is urgently needed to generate profession‐specific data. Organisations and senior managers should actively promote restorative clinical supervision across radiography, allocate protected time for training and sessions and ensure staff at all levels understand how to access it. Empowering internationally educated and minority staff into the professional advocate role, ideally including those with lived experience of international migration and facilitating systematic evaluation of outcomes should be explicit organisational priorities. Professional advocates, having completed A‐EQUIP education, should offer both individual and group restorative clinical supervision and maintain ongoing CPD that includes cultural competence, with regular supervision of supervisors to sustain quality. Professional bodies should update guidance for AHPs, develop radiography‐specific educational resources and commission and support primary research into the experiences of internationally educated radiographers and the feasibility and effectiveness of A‐EQUIP implementation in diagnostic radiography settings.

## 6 Conclusions

Retaining diagnostic radiographers is essential to the sustainability of diagnostic services both in the UK and globally. For internationally educated radiographers, the occupational stressors common to the profession are compounded by the significant personal and professional challenges of migration. This is a combination that, without structured support, too often ends in attrition.

The radiography profession has been slow to engage with restorative clinical supervision: unlike nursing and midwifery, where it is embedded in professional expectations, it remains optional and poorly understood. The A‐EQUIP model offers a coherent and adaptable framework for addressing this gap, with the restorative function offering the most immediately evidenced route to supporting internationally educated radiographers. Significant evidence gaps remain, particularly regarding long‐term effectiveness in radiography and the specific experiences of internationally educated radiographers; closing them must now be a research priority.

## Funding

The authors have nothing to report.

## Disclosure

Permission to reproduce material from other sources: Figure 2 (The A‐EQUIP Model) is adapted from NHS England, Professional Nurse Advocate A‐EQUIP Model: A Model of Clinical Supervision for Nurses (Version 2, September 2023), available at https://www.england.nhs.uk/long‐read/pna‐equip‐model‐a‐model‐of‐clinical‐supervision‐for‐nurses/. Contains public sector information licensed under the Open Government Licence v3.0. The source is cited in the manuscript [38].

## Ethics Statement

This work did not require review by an ethics committee as it does not constitute primary research involving human participants, data collection or interventions. All information used was pre‐existing, published literature and did not involve identifiable personal data; therefore, formal ethical approval was not applicable for this commentary.

## Consent

The authors have nothing to report.

## Conflicts of Interest

The authors declare no conflicts of interest.

## Data Availability

Data sharing not applicable to this article as no datasets were generated or analysed during the current study.
